# Benign yet deceptive: A rare spindle-cell neoplasm mimicking a cystic tumor - A case report on gastric schwannoma

**DOI:** 10.1016/j.ijscr.2025.111708

**Published:** 2025-07-23

**Authors:** Reshu Karki, Sayujya Khanal, Prabir Maharjan

**Affiliations:** a Kathmandu Medical College and Teaching Hospital, Kathmandu, Nepal; b Department of Gastrointestinal and General Surgery, Kathmandu Medical College and Teaching Hospital, Kathmandu, Nepal

**Keywords:** Gastric neoplasms, Gastrointestinal stromal tumors, Immunohistochemistry, Neurilemmoma, Schwann cells, Schwannoma

## Abstract

**Introduction and importance:**

Gastric schwannomas are uncommon, benign tumors originating from Schwann cells within the enteric nerve plexus, comprising 6.3 % of mesenchymal tumors in the stomach and 0.2 % of all gastric neoplasms. Due to their submucosal origin and spindle-cell structure, they can resemble gastrointestinal stromal tumors (GISTs), making immuno-histochemical analysis crucial for accurate diagnosis.

**Case presentation:**

A 61-year-old woman presented with a progressively enlarging abdominal mass over the past five months, with no pain, nausea, or gastrointestinal bleeding. Physical examination revealed a firm, mobile mass measuring 8 × 7 cm^2^. Routine investigations were inconclusive. Upper gastrointestinal endoscopy showed gastric erosions but no concerning mass. A contrast-enhanced CT scan identified a well-defined, predominantly cystic mass arising from the greater curvature of the stomach.

**Clinical discussion:**

The patient underwent laparoscopic wedge resection in which the tumor and a portion of the adjacent stomach were resected and histopathological examination confirmed a well-defined spindle-cell tumor with mild nuclear pleomorphism. Immunohistochemistry showed strong positivity for S-100 and Sox-10 proteins, confirming gastric schwannoma. The patient recovered well after surgery with no complications. She was discharged in stable condition, and follow-up evaluations were unremarkable in the short term.

**Conclusion:**

Gastric schwannomas are slow-growing, benign tumors with an excellent prognosis. Differentiation from gastrointestinal stromal tumors is crucial for proper treatment planning. Complete surgical resection is curative, and recurrence is rare, emphasizing the importance of accurate diagnosis for optimal patient care.

## Highlights

• Rare, benign tumors originating from Schwann cells within the enteric nerve plexus
• Tumors closely resemble gastrointestinal stromal tumors (GISTs), mandating immuno-histochemical analysis for accurate diagnosis
• Contrast CT and histopathology are key to differentiating gastric schwannomas from other gastric tumors.
• Surgical resection remains definitive treatment with a good prognosis and no reported cases of recurrence post complete tumor removal

## 1 Introduction

Schwanommas are rare gastrointestinal (GI) mesenchymal tumors, usually benign and well circumscribed, consisting of a clonal population of Schwann cells which are prone to cystic and degenerative changes [1,2]. The majority of the gastric mesenchymal tumors are stromal, with schwannomas accounting for 6.3 % of all mesenchymal tumors and 0.2 % of all gastric tumors [1]. Having the potential of arising from any nerve from the body, these are usually attached to peripheral nerves, and most commonly are located in the soft tissues of the head and neck, or the flexor surfaces of extremities [3,4].

Schwannomas in the GI tract are usually rare, and have a predilection for the stomach (60–70 % of cases), followed by the colon and rectum [[5], [6], [7], [8], [9]]. In contrast to gastric gastrointestinal stromal tumors (GIST), which have malignant potential, schwannomas behave benignly and there has been no recurrence, metastasis, or tumor-related mortality reported after curative resection. [1,4,5]

Diagnostic modalities include upper GI endoscopy (UGIE), computed tomography (CT) scan, magnetic resonance imaging (MRI), ultrasonography (USG), endoscopic ultrasonography (EUS), and upper GI barium study. However, all these investigations provide only limited information about the tumor. The definitive diagnosis of gastric schwannoma is determined only after histopathological and immune-histochemical examination [10].

The existence of schwannoma as a primary gastrointestinal tumor was debated until a case series presented by Daimaru et al. with 24 well-documented cases were presented [11]. The aim of this case report is to document this rare tumor of the GI tract since there have been very few cases reported so far, and highlighting its significance to be recognized as a differential diagnosis.

## 2 Case presentation

A 61 years lady presented to the outpatient department with a history of swelling in the abdomen for 5 months, which was acute in onset, gradually progressive, fixed, firm, not associated with pain, nausea and vomiting. The patient gave no history of fever, hematemesis, malena, jaundice diarrhea, constipation, significant weight loss or anorexia. She had no significant past medical or surgical history and unremarkable family history. She had a history of tobacco consumption and alcohol consumption, which she discontinued 30 years back.

The patient was well oriented to time, place and person, was hemodynamically stable and had stable vitals. Systemic examination revealed a single intra-abdominal non-tender globular mass measuring 8*7 cm^2^, mobile in horizontal direction, firm in consistency, with a smooth surface and regular margins extending from the epigastrium up to the hypogastrium.

**Fig. 1:**
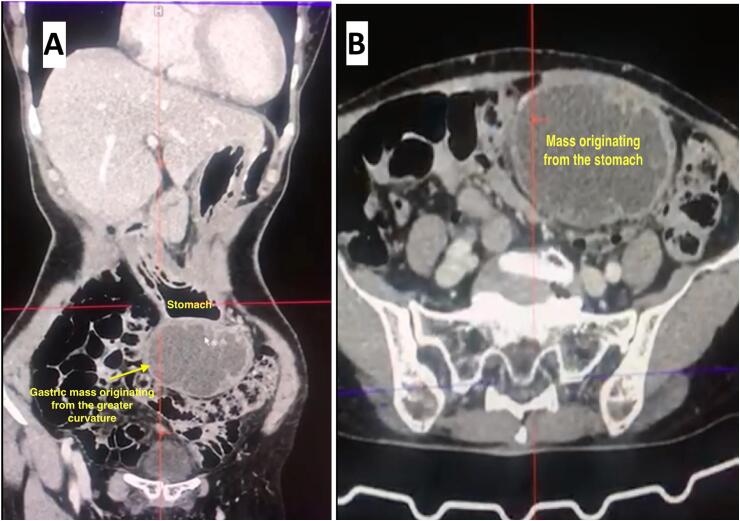
(A, B) A well-defined exophytic and predominantly cystic mass arising from the greater curvature of the stomach, measuring approximately 6.19*5.63*8.31 cm^3^, extending infero-laterally with peripheral and septal enhancement, being supplied by a branch of the celiac artery.

Routine blood and urine tests were performed, which were within normal limits. Clinical differential diagnosis of either a mesenteric cyst, gastric, or enteric mass was discussed. An upper GI endoscopy failed to reveal any obvious mass or lesions except for few small antral erosions. For a better diagnostic approach, a contrast enhanced CT (CECT) of abdomen and pelvis was requested which revealed a well-defined exophytic and predominantly cystic mass arising from the greater curvature of the stomach, measuring approximately 6.19*5.63*8.31 cm^3^, extending infero-laterally with peripheral and septal enhancement, being supplied by a branch of the celiac artery, highly suggestive that the mass was a neoplastic lesion of the stomach (Fig. 1).

**Fig. 2:**
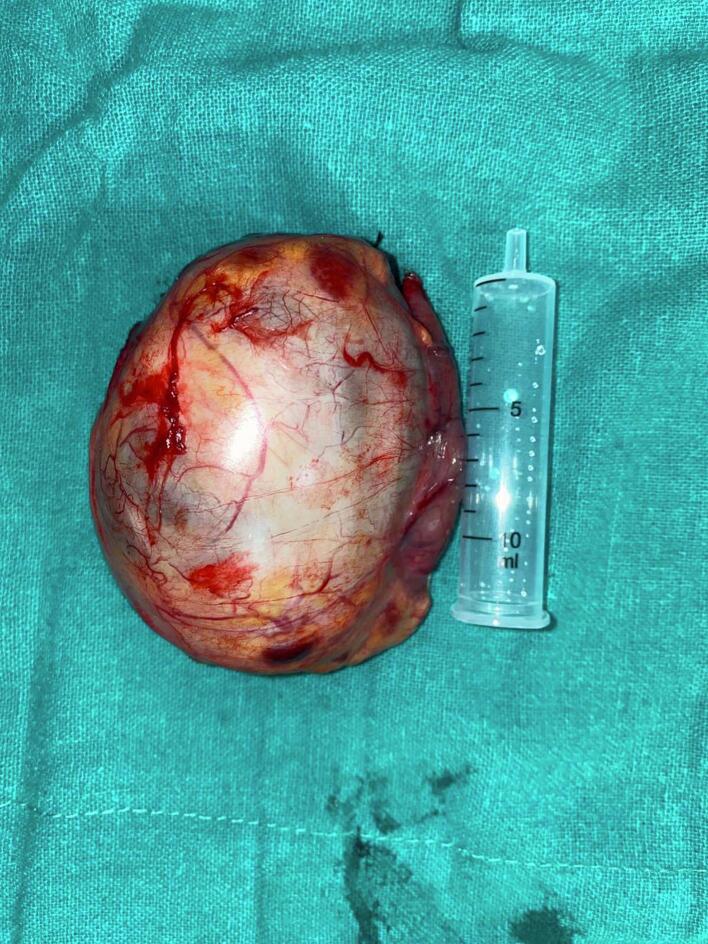
The resected portions of the stomach and attached mass measured 8 × 6 cm^2^, with one cm resection margin.

The patient was advised for surgical resection of the tumor. Considering the size of the mass and her thin build, wedge resection of the stomach was performed laparoscopically after routine pre-operative evaluation. The resected portions of the stomach and attached mass measured 8 × 6 cm^2^, with one cm resection margin (Fig. 2).

**Fig. 3:**
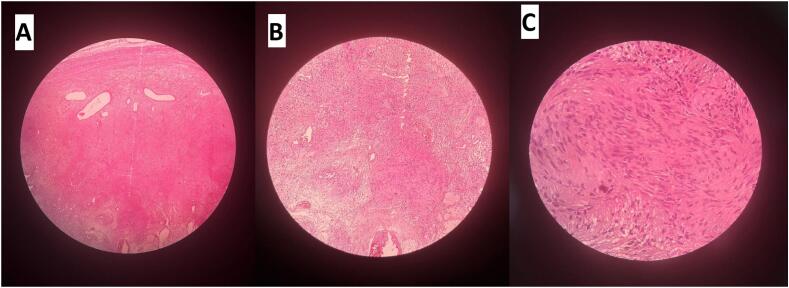
(A) 4× image: Hypercellular and hypocellular areas. (B) 10× image: Hypercellular areas with verocay bodies and hypocellular areas with myxoid changes. (C) 40× image: Mild nuclear pleomorphism. Nuclei are round to oval to spindle shaped with coarse chromatin.

Histopathologic correlation of the mass revealed a yellowish, gelatinous mass with hemorrhagic areas and cystic changes, which is infrequent. Microscopic examination of the same revealed gastric mucosa lined by foveolar epithelium, sub-serosa housing a well circumscribed tumor mass composing of interlacing fascicles of spindle-shaped tumor cells exhibiting mild nuclear pleomorphism. There were hypercellular areas with verocay bodies and hypocellular areas with myxoid changes. Nuclei were round to oval to spindle shaped with coarse chromatin. Sections from the resected margins or lymph nodes were uninvolved by the tumor (Fig. 3).

Following histopathological examination, a customized immunohistochemistry test relying upon immunoreactivity of the tumor cells with S 100 and Sox 10 was performed for confirmation, which favored the lesion to be mesenchymal with neural origin, a gastric Schwannoma. Other tumor markers including CA 10–9, CEA, NSE were not checked in this patient due to financial constraints.

The patient was stable post-operatively, thus was shifted to the surgical intensive care unit (SICU) and was kept under observation for the next 24 h. Her post-operative stay was uneventful with no complications, and she was shifted to the surgical ward the next day and discharged on the fifth day of admission.

Patient was followed up after a week and a month of surgery. The patient has no issues till date and planned for follow-up after six months and then annually. The patient will undergo UGIE and USG evaluation in the next follow up.

## 3 Discussion

Schwann cells are glial cells whose precursors arise from neural crest cells and are responsible for generation of myelin sheath and provision of trophic support to the neurons they ensheathe [12]. Among the peripheral nerve sheath tumors, schwannomas, also known as neurinoma and neurilemmoma are the most common ones among neurofibromas, perineuromas, granular cell tumors and malignant nerve sheath tumors [2]. GI schwannomas originate from Schwann cells of nerve sheath, more frequently from the Auerbach's plexus than the Meissner's plexus [13].

Gastric schwannomas occur more frequently in the third to fifth decade of life and are usually solitary tumors arising from the fundus, body or antrum of the stomach [7,11,[14], [15], [16], [17], [18]]. However, they can occur in children and rarely be malignant [19]. These tumors occur at any age, but generally between the ages of 40 and 60, with an average of 58 years and a slight female predominance as seen from various studies [[20], [21], [22]].

GI schwannomas were described by Daimaru et al. highlighting the origin of the tumors from the muscularis propria of the stomach in 23 cases and in the ascending colon in one case from the 24 cases they reviewed. The possibility of Myenteric plexus origin of the tumor was indicated by positive immunoreactivity for glial fibrillary acidic protein (GFAP). Positive immunostaining for S-100 protein, Leu 7 antigen, and laminin supported the schwannian nature of these tumors. They proposed that these tumors be designated as benign schwannoma of the GI tract [11].

The most common presenting symptom has been stated as abdominal pain or discomfort, but not GI bleeding [23]. Clinically, it remains asymptomatic for years in 30 to 50 % of cases, and even though a patient may present with diverse presentations, specific signs are usually absent. Its discovery is often incidental during imaging, endoscopy, or surgery performed for another pathology [13].

On radiographic examination, gastric schwannomas appear as submucosal tumors with CT features of homogenous and well demarcated masses. EUS findings are usually consistent with homogenous submucosal masses contiguous with muscularis propria [24]. They usually appear as spherical, ovoid or multi-lobular solid masses adjacent to gastric wall, which may undergo cystic degeneration or calcification, with the latter being uncommon [13]. EUS due to its capability of allowing deeper biopsies than conventional endoscopy proves to be an efficient technique for exploration of the mass, which usually appears rounded, located at the muscular or submucosal layer, hypoechoic and homogenous. However these signs may also be found in GISTs, thus mandating immunochemistry [25]. Microscopically they consist of interlacing bundles of spindle cells and on immunochemistry, positivity for S-100 protein in the tumors are highly confirmative for schwannoma [24]. MRI with multi-planar imaging capability may be adjuvant to determining the exact location and extent of the tumor, additionally giving information on displacement of surrounding organs or vessels which may be crucial for surgical planning and overall outcome of the intervention. MRI appearance are typically sharply demarcated and strongly enhancing, with low to medium signal intensity on T1 weighted images and high signal intensity on T2 weighted images [13]. Endoscopically guided fine-needle aspiration is not routine as recommended by the National Comprehensive Cancer Network (NCCN) for resectable submucosal tumors (SMT) due to the risk of tumor rupture and spread, which is associated with poor prognosis [26].

The most common differential diagnosis of gastric schwannomas are GISTs. However, most cases are diagnosed post-operatively via histopathology and immunochemistry, which is a diagnostic limitation. The pattern of S-100 protein immunostaining differed from that seen in cases of gastrointestinal stromal tumors associated with von Recklinghausen's neurofibromatosis or that noted in cases of conventional leiomyomas [11]. Clinically, peculiar features like growth of the two (*exo*-luminal v/s *endo*-luminal-combined respectively), radiological appearance (rounded and homogenous v/s irregular and heterogenous respectively) and presence of intra-tumor necrosis (rare v/s frequent respectively) may be a strong clue for radio-clinical differentiation of the two [27].

Complete surgical resection is widely considered to be a curative treatment for GS, and laparoscopic or open approaches for wedge resection, subtotal gastrectomy or near total resection, and total gastrectomy are the treatments of choice [[28], [29], [30], [31], [32], [33], [34]]. Even though not backed by large-sample multicenter studies on the efficacy, safety and outcomes, minimally invasive surgical approaches including endoscopic submucosal tunneling resection, endoscopic enucleation and endoscopic full-thickness resection with or without laparoscopic assistance are frequently used as diagnostic and interventional modalities for GS [[35], [36], [37], [38], [39], [40], [41], [42], [43]].

The management of gastric schwannomas primarily involves surgical resection, which has traditionally been the mainstay treatment. However, recent studies have highlighted the role of endoscopic resection as a viable alternative, particularly for smaller tumors. Endoscopic resection techniques, such as endoscopic submucosal excavation (ESE) and endoscopic full-thickness resection (EFTR), have been shown to be effective and safe, with favorable long-term outcomes [[44], [45], [46], [47]]. These techniques are associated with shorter operative times, less blood loss, and lower medical costs compared to surgical resection, making them attractive options for tumors smaller than 3 cm without signs of malignancy [44,48].

Hu et al. (2017) further supported the role of ER, highlighting the utility of endoscopic ultrasound (EUS) in the preoperative assessment of GS. EUS can help differentiate GS from other submucosal tumors, although it remains challenging to distinguish GS from gastrointestinal stromal tumors (GISTs) based solely on imaging. The study reported successful complete endoscopic resection in all patients, with no complications or recurrence during follow-up, underscoring ER as an effective and safe treatment modality for GS [47].

The study by Tao et al. [49] from China highlighted the challenges in distinguishing GS from GISTs preoperatively and noted the benefits of laparoscopic resection, which is associated with less blood loss and shorter hospital stays compared to open surgery. This study reinforced the notion that GS is often benign, with excellent long-term outcomes following resection [49].

Overall, the global literature on gastric schwannomas indicates a trend towards utilizing minimally invasive techniques, such as endoscopic and laparoscopic resection, for the management of these tumors. The choice of treatment modality is influenced by tumor size, location, and the availability of expertise in advanced endoscopic techniques.

Even though rare, gastric schwannoma should be included in differential diagnoses of intramural or exophytic gastric masses when imaging findings reveal a well-defined solid mass adjacent to the stomach, or in the differentials of gastric submucosal tumors [13,23]. A 2015 study by Hong et al. reviewed 137 cases of GS and reported no recurrence or metastasis during a follow-up period ranging from 1 to 336 months. It was concluded that benign GS typically does not recur, and frequent morphologic assessment during follow-ups are unnecessary [50]. However, a study by Choi et al. reported a mean tumor doubling time of nearly 5 years based on CT scans from a series of follow-up images [51].

Excellent prognosis of GS indicates that post-therapeutic follow up be done simply clinically, and the need of a radiologic or morphologic assessment may be obsolete. Due to its benign and slow-growing nature overlapped with non-specific clinical, radiological, or endoscopic features, diagnosis may be delayed or difficult preoperatively.

## 4 Conclusion

Gastric schwannomas are rare, benign tumors originating from Schwann cells, predominantly found in the stomach. They are often asymptomatic and may be discovered incidentally during imaging or endoscopic procedures. The definitive diagnosis of gastric schwannoma relies on immune-histochemical staining, with positivity for S-100 protein being a key diagnostic marker, while other markers such as CD117, CD34, SMA, desmin, and DOG-1 are typically negative.

Surgical resection remains the standard treatment for gastric schwannomas, offering excellent long-term outcomes with minimal recurrence risk. Laparoscopic surgery is preferred for small to moderate-sized tumors due to its association with less blood loss and shorter postoperative hospital stays compared to open surgery. ER has emerged as a viable alternative, particularly for tumors smaller than 3 cm, providing similar safety and efficacy to surgical resection but with shorter operative times and lower medical costs. Techniques such as endoscopic submucosal excavation ESE and EFTR have demonstrated favorable outcomes, including high complete resection rates and low recurrence during follow-up.

Overall, gastric schwannomas have a favorable prognosis following resection, whether surgical or endoscopic, with minimal risk of recurrence or metastasis. The choice between surgical and endoscopic resection may depend on tumor size, location, and the presence of symptoms, with ER being particularly suitable for smaller tumors without signs of malignancy.

## 5 Methods

This case has been reported in line with the SCARE criteria [52].

## CRediT authorship contribution statement

All authors contributed to the study's conception and design. Material preparation and data collection were performed by R.K., S.K., P.M. The first draft was written by R.K. and all authors commented on previous versions of the manuscript. All authors read and approved the final manuscript.

## Informed consent

The patient and respective legal guardians were informed about the publication and provided informed consent before reporting.

## Consent for publication

Written informed consent was obtained from the patient herself for the publication of this case report and accompanying images. A copy of the written consent is available for review by the Editor-in-Chief of this journal on request.

## Ethical approval

Our institution does not require ethical approval for reporting individual cases.

## Guarantor

Reshu Karki.

## Sources of funding

No funding was received from any internal or external sources.

## Declaration of competing interest

The author(s) declared no potential conflicts of interest with respect to the research, authorship, and/or publication of this article.
